# Association Between Physical Activity, Body Mass Index, and Aerobic Capacity in Periurban Adolescents

**DOI:** 10.3390/ijerph23060806

**Published:** 2026-06-17

**Authors:** Fabian Sepúlveda, Ana Peñata-Taborda, Osnamir Bru-Cordero, Leily Montoya-Álvarez, Alicia Humanez-Álvarez

**Affiliations:** 1 Programa de Maestría en Epidemiología de la, Universidad del Sinú–Elías Bechara Zainúm, Montería 230001, Colombia; edgarfrodriguez@unisinu.edu.co; 2 Grupo de Investigación Biomédica y Biología Molecular, Universidad del Sinú–Elías Bechara Zainúm, Montería 230001, Colombia; investigador01gibm@unisinu.edu.co; 3 Dirección Académica, Universidad Nacional de Colombia Sede de La Paz, Cesar 200017, Colombia; oebruc@unal.edu.co; 4 Grupo de Investigación MOSABI, Universidad del Sinú–Elías Bechara Zainúm, Montería 230001, Colombia; leilymontoya@unisinu.edu.co

**Keywords:** cardiorespiratory fitness, nutritional status, periurban health, behavioral determinants and multivariable analysis

## Abstract

**Highlights:**

**Public health relevance—How does this work relate to a public health issue?**

- This study provides evidence from a periurban population, a context undergoing rapid territorial and epidemiological transitions that is rarely examined

**Public health significance—Why is this work of significance to public health?**

- Physical activity showed an independent association with aerobic capacity, whereas BMI did not after adjustment, highlighting differential contributions of behavioral versus anthropometric factors.

**Public health implications—What are the key implications or messages for practitioners, policy makers, and/or researchers in public health?**

- Strategies promoting sustained engagement in physical activity may be particularly relevant in territorially transitioning environments.

**Abstract:**

Adolescence is a critical window for health behavior consolidation, yet the combined influence of physical activity level (PAL) and the body mass index (BMI) on aerobic capacity remains understudied, especially in transitioning periurban environments. This study examined the association between PAL, BMI, and aerobic capacity in adolescents from Montería, Colombia. A cross-sectional study was conducted among 112 adolescents (aged 12–17 years). Aerobic capacity was assessed using the 20 m shuttle run test (Course Navette), and PAL was measured via the validated Assessment of Physical Activity Levels Questionnaire (APALQ), following standard fitness assessment protocols. Multivariable linear regression models were utilized to estimate independent associations, adjusting for age and sex. The multivariable model was significant (F = 8.45; *p* < 0.001), explaining 21% of the variance in aerobic capacity (adjusted R^2^ = 0.21). PAL was positively and independently associated with aerobic capacity (B = 0.22; 95% CI: 0.05–0.38; *p* = 0.010), regardless of BMI. While age showed a positive association (B = 0.09; *p* = 0.032) and sex was inversely associated (B = −0.39; *p* < 0.001), BMI did not emerge as an independent predictor in the adjusted model (B = −0.04; *p* = 0.080). Aerobic capacity in adolescents is more consistently explained by behavioral factors (what they “do”) than by anthropometric status (what they “weigh”). These findings support a paradigm shift in pediatric public health, prioritizing high-intensity movement overweight control to improve cardiorespiratory fitness in transitioning urban territories.

## 1. Introduction

The sustained rise in chronic non-communicable diseases (NCDs) has led to a heightened focus in the scientific community on the early-life determinants of cardiometabolic health [1,2]. Adolescence is a critical developmental period in this context. It is characterized by significant biological maturation and the consolidation of behavioral patterns that of-ten persist into adulthood [3]. Among these, physical activity and sedentary behaviors have emerged as key modifiable factors shaping current and future health trajectories. A decline in physical activity levels during adolescence, accompanied by increasing sedentary time and marked sex differences, has been widely documented and linked to unfavorable changes in physical fitness and body composition [4]. Collectively, these trends underscore adolescence as a pivotal period for understanding the early structuring of functional health.

Cardiorespiratory fitness, defined as the maximal capacity of the cardiovascular, respiratory, and muscular systems to oxidize oxygen, is typically measured through two methods: maximal oxygen uptake (VO_2_max) and standardized field tests [5]. In adolescents, there is a consistent association between aerobic capacity and metabolic efficiency, as well as cardiovascular health. Conversely, lower levels of aerobic capacity are associated with higher adiposity, reduced physical performance, and less favorable cardiometabolic profiles [6]. Aerobic capacity is not merely a physiological attribute; rather, it is a significant indicator of functional health. This is because it captures the interplay between behavioral and biological processes during a critical stage of development.

The body mass index (BMI) remains a widely used epidemiological indicator of nutritional status in youth, despite its known limitations in distinguishing body composition [7]. Both elevated and low BMI values have been associated with compromised aerobic performance, though through distinct mechanisms [8]. Excess body mass imposes greater mechanical and cardiovascular demands during physical exertion, whereas low BMI may reflect insufficient nutritional reserves affecting muscle development and endurance [9]. In Latin American countries, including Colombia, the coexistence of undernutrition and overweight or obesity, commonly referred to as the double burden of malnutrition (DBM), is well documented among adolescent populations [10,11]. This dual nutritional landscape complicates the interpretation of fitness patterns, as adolescents with markedly different physiological profiles may coexist within the same school and community environment [12].

Physical activity level (PAL) is closely linked to both BMI and aerobic capacity, yet its distribution during adolescence is heterogeneous [13]. Although declines in PAL with increasing age and consistently lower levels among girls have been widely documented, the extent to which these factors interact rather than operate independently remains insufficiently understood. Moreover, physical activity behaviors are not shaped solely by biological characteristics but are embedded within broader environmental and territorial contexts that structure daily opportunities for movement [12].

Periurban environments represent a distinct and understudied setting in this regard. As transitional spaces between rural and urban systems, periurban areas are subject to rapid land-use changes, altered mobility patterns, and progressive loss of open or agricultural spaces [14]. Such changes may constrain habitual physical activity while simultaneously reshaping dietary practices and social routines. Rather than merely serving as geographic intermediaries, periurban environments may generate unique configurations of behavioral and anthropometric risk. Although contrasts between rural and urban adolescent populations have been described, periurban contexts are frequently subsumed within these broader categories, limiting understanding of how territorial transition jointly influences physical activity, nutritional status, and aerobic capacity [15,16].

Despite growing evidence linking physical activity, BMI and aerobic capacity in adolescents [17], these factors have often been examined independently or within predominantly urban populations. Less attention has been given to how they jointly shape variability in aerobic capacity, particularly among adolescents living in periurban environments undergoing rapid territorial and lifestyle transition. Understanding whether these relationships operate uniformly or differ according to anthropometric context remains an important unresolved question in adolescent health research. Accordingly, this study examined the joint association between PAL, BMI and aerobic capacity in adolescents attending a public school in a periurban area of Montería, Colombia, with the aim of characterizing how these factors relate within this specific environmental setting.

## 2. Materials and Methods

### 2.1. Participants and Estudy Design

A cross-sectional analytical study was conducted to examine the association between PAL, BMI and aerobic capacity among adolescents attending a public educational institution located in a periurban area of Montería, Córdoba, Colombia. The study’s sample population comprised 112 adolescents, ranging in age from 12 to 17 years. Participants were recruited using a non-probabilistic convenience sampling approach based on eligibility criteria and school availability.

The inclusion criteria encompassed the following: (i) the participant must be within the established age range, (ii) written informed consent from parents or legal guardians must be provided, and (iii) adolescent assent must be obtained. Exclusion criteria encompassed medical conditions that imposed limitations on physical exertion, recent surgical interventions, ongoing clinical treatments, or any physical or cognitive limitations that precluded participation in field assessments.

A comprehensive array of sociodemographic, anthropometric, and behavioral variables was systematically collected, encompassing age, sex, BMI, PAL and aerobic capacity. The study protocol was approved by the Research Ethics Committee of the Faculty of Health Sciences at Universidad del Sinú–Elías Bechara Zainúm (Act No. 002, 15 April 2024). The research was conducted in accordance with the principles of the Declaration of Helsinki and national regulations for minimal-risk research. Prior to data collection, authorization was obtained from the relevant school authorities, and written informed consent and assent were secured.

### 2.2. Anthropometric Measurement

Anthropometric measurements were obtained following standardized procedures recommended by the World Health Organization (WHO) and national guidelines established in Resolution 2465 of 2016 issued by the Colombian Ministry of Health. Body weight was measured using a calibrated digital scale (B04, Femmto, Shenzhen, China; precision 0.1 kg), with participants barefoot and wearing light clothing. Height was measured using a portable stadiometer (HM200P, Charder Medical, Taichung, Taiwan), ensuring proper anatomical positioning according to the Frankfurt plane.

Based on weight and height measurements, BMI was calculated using the Quetelet Index (BMI = weight [kg]/height^2^ [m^2^]). BMI values, together with participants’ exact age (expressed in years and months), were used to classify the nutritional status of children and adolescents under 18 years of age, according to age- and sex-specific percentiles established in the WHO growth and Colombian health regulations, categorizing participants into the corresponding nutritional status groups.

### 2.3. Aerobic Capacity (Course Navette Test)

Aerobic capacity was assessed using the 20 m shuttle run test (Course Navette), an internationally validated field test for estimating cardiorespiratory fitness in children and adolescents [18]. Participants ran repeatedly between two lines set 20 m apart, following paced auditory signals with progressively increasing speed, beginning at 8.5 km/h and increasing by 0.5 km/h each minute [19,20]. The test was terminated when the participant failed to reach the line on two consecutive occasions or voluntarily stopped due to exhaustion. The final completed stage was recorded as the performance indicator and subsequently classified according to age- and sex-specific reference values. All assessments were conducted in the school, setting under standardized conditions and supervised by trained personnel to ensure procedural consistency.

### 2.4. Physical Activity Levels Questionnaire (APALQ)

Physical activity was assessed using the APALQ, a previously validated instrument for evaluating habitual physical activity in adolescents [21]. The questionnaire captures participation in organized sports, non-organized physical activities, engagement in physical education classes, weekly vigorous activity, and involvement in competitive sports.

Item scores were summed to obtain a total score, and participants were classified as sedentary (5–10 points), moderately active (11–16 points), or very active (≥17 points) according to established cut-off values. In the present study, the instrument’s internal consistency and factorial structure were examined to confirm its psychometric adequacy for the study population.

### 2.5. Statistical Analysis

Categorical variables are presented as percentages with Wilson 95% confidence intervals (CIs), whereas continuous variables are reported as mean ± standard deviation (SD) or median with interquartile range (IQR), according to distributional properties. Between-group comparisons were performed using Student’s *t*-test or the Mann–Whitney U test, as appropriate. Associations between categorical variables were examined using chi-square tests (2 × k tables), and sex-based differences in proportions were assessed with two-sided z tests using Wilson confidence intervals. The psychometric performance of the APALQ was evaluated using Cronbach’s alpha, composite reliability, and confirmatory factor analysis based on diagonally weighted least squares estimation for ordinal items, reporting robust chi-square (χ^2^), CFI, RMSEA, and SRMR. Missing data (<10%) were addressed using complete-case analysis, and robustness was evaluated through multiple imputation procedures (m = 20). Spearman rank correlation coefficients were calculated to examine bivariate associations between APALQ items and aerobic capacity, given the ordinal nature of the variables.

Multivariable associations were examined using linear regression models. The dependent variable was the aerobic capacity classification derived from the 20 m shuttle run test, categorized into three ordered levels (Needs Improvement, Acceptable, Outstanding) according to age- and sex-specific normative standards. For analytical purposes, categories were coded as an ordinal score (1–3) and treated as an approximately continuous outcome. Independent variables included physical activity level (APALQ), BMI (kg/m^2^), age, and sex. Unstandardized regression coefficients (B), 95% confidence intervals, and model fit statistics were reported. Additionally, an exploratory unsupervised k-means clustering analysis was conducted on standardized BMI, physical activity and aerobic capacity variables to identify potential multivariate fitness profiles. All statistical tests were two-sided with a significance level of α = 0.05 and were performed using R software (version 4.3.3; R Core Team, R Foundation for Statistical Computing, Vienna, Austria).

## 3. Results

### 3.1. Sociodemographic and Anthropometric Profile of the Study Population

A total of 112 adolescents were included in the analysis, comprising 53 females (47.3%) and 59 males (52.7%). The mean age of the study population was 14.70 ± 1.29 years, with a median of 15 years (Q1–Q3: 14–16) and an age range of 12–17 years. Age distributions were comparable between females and males, with no statistically significant differences observed (*p* = 0.684) (Table 1).

Regarding educational level, participants were predominantly enrolled in 8th and 9th grade (32.14% each), followed by 11th grade (23.21%) and 10th grade (12.5%). Sex-specific distributions showed a higher proportion of males in lower grades and a greater representation of females in upper grades. These differences are reported descriptively, as grade level comparisons by sex were not subjected to inferential testing. Anthropometric characteristics showed a mean body weight of 51.77 ± 8.83 kg in the total sample. Although males had a higher mean body weight than females (52.95 ± 9.81 kg vs. 50.46 ± 7.48 kg), the difference was not statistically significant (*p* = 0.138). Mean height was 162.10 ± 8.74 cm, with males being significantly taller than females (166.28 ± 8.61 cm vs. 157.45 ± 6.20 cm; *p* < 0.001).

The mean BMI was 19.67 ± 2.82 kg/m^2^ for the overall population. Females presented significantly higher BMI values compared with males (20.33 ± 2.58 kg/m^2^ vs. 19.08 ± 2.92 kg/m^2^; *p* = 0.004). According to the BMI-for-age classification established by Resolution 2465 of 2016 of the Colombian Ministry of Health, most participants were classified as having normal weight (70.54%), followed by thinness (11.61%), risk of thinness (10.71%), overweight (6.25%), and obesity (0.89%) (Table 1).In addition, BMI category distribution differed significantly by sex (permutation-based χ^2^ test; *p* < 0.05).

### 3.2. Psychometric Properties and Sex-Based Classification of the APALQ

Given that the APALQ constitutes the primary instrument for assessing physical activity in this study, its psychometric properties were evaluated in the study population to ensure the reliability and structural adequacy of the scores prior to conducting comparative analyses.

The APALQ demonstrated adequate internal consistency in the study population (Cronbach’s α = 0.78; composite reliability = 0.84), supporting the use of the total score in subsequent analyses. Confirmatory factor analysis estimated using diagonally weighted least squares (DWLS) for the four ordinal items of the questionnaire (Ask_1, Ask_2, Ask_4, and Ask_5), supported a unidimensional structure. Standardized factor loadings were of moderate to high magnitude (0.52–0.91), and response thresholds were ordered, indicating appropriate category functioning.

According to the validated APALQ classification, significant differences in physical activity profiles were observed between sexes, as detailed in Table 2. Females exhibited a higher prevalence of sedentary behavior (49.1%; 95% CI: 36.1–62.1) than males (16.9%; 95% CI: 9.5–28.5; two-sided proportion test, *p* < 0.001). Conversely, males were more frequently classified as moderately active (62.7%; 95% CI: 50.0–73.9), while only 35.8% (95% CI: 24.3–49.3) of females were classified in this category (*p =* 0.004). The proportion of adolescents classified as very active was similar across sexes, with 20.3% of males and 15.1% of females identified as such (*p* = 0.469). Overall, the distribution of APALQ categories differed significantly between males and females, as indicated by χ^2^(2) = 13.41, *p* = 0.001.

### 3.3. Aerobic Capacity According to Sex

The distribution of aerobic capacity levels in the study population is shown in Table 3. Overall, adolescents were classified across three categories: Needs Improvement, Acceptable, and Outstanding. The distribution of aerobic capacity categories differed significantly by sex (χ^2^(2) = 20.42, *p* < 0.001).

A markedly higher proportion of females was classified in the Needs Improvement category (92.5%; 49/53) compared with males (54.2%; 32/59), with this difference reaching statistical significance (two-sided proportion test, *p* < 0.001). Conversely, males were more frequently classified in the Acceptable (30.5% vs. 5.7%; *p* ≤ 0.001) and Outstanding (15.3% vs. 1.9%; *p* = 0.013) categories. Overall, sex-related differences in aerobic capacity were concentrated in the middle and upper performance categories, with males showing a higher representation in these tiers.

### 3.4. Distribution of Aerobic Capacity Across BMI Categories

Table 4 presents the joint distribution of aerobic capacity and BMI classification. In the total sample, a majority of adolescents (72.3%) were classified as having a “Needs Improvement” level of aerobic capacity, regardless of their BMI category. When examining distribution by BMI categories, those with a normal weight represented the highest proportion across all aerobic capacity levels. Specifically, 76.5% of adolescents in the normal weight group were classified as “Needs Improvement,” followed by 57.1% in the “Acceptable” category and 50.0% in the “Outstanding” category. Participants classified as underweight, at risk of being underweight, and overweight also predominantly fell into the “Needs Improvement” category. Notably, the obesity category included only one participant, who was also classified as needing improvement. The data indicates a predominance of lower aerobic capacity levels across all BMI categories, with fewer individuals categorized as Acceptable or Outstanding.

### 3.5. Correlation Between APALQ Items and Aerobic Capacity

**Figure 1:**
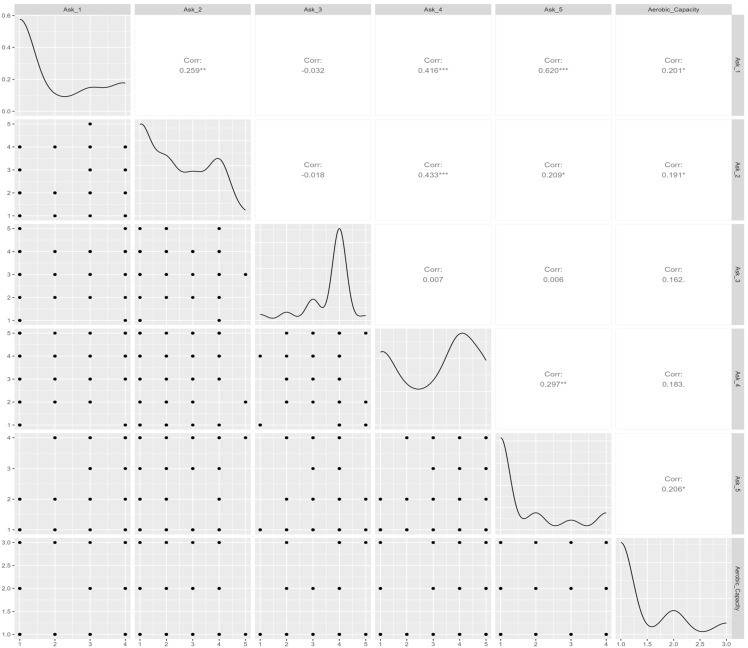
Spearman correlations between APALQ items and aerobic capacity. The matrix displays pairwise Spearman correlation coefficients (ρ) between APALQ items (Ask_1–Ask_5) and aerobic capacity. Scatterplots are shown in the lower panels, density plots on the diagonal, and correlation coefficients in the upper panels. Significance levels are indicated as follows: * *p* < 0.05; ** *p* < 0.01; *** *p* < 0.001.

Spearman correlation analyses were conducted to examine the bivariate associations between the APALQ items and aerobic capacity, considering the ordinal nature of the variables (see Figure 1). Bivariate analyses revealed positive correlations between several physical activity dimensions and aerobic capacity (Figure 1). The strongest association was observed for participation in competitive sports (ρ = 0.62; *p* < 0.001), followed by engagement in organized sports outside the school setting (ρ = 0.42; *p* < 0.001) and weekly vigorous physical activity (ρ = 0.30; *p* < 0.01). Non-organized physical activity showed a weaker but significant correlation (ρ = 0.26; *p* < 0.01), whereas physical activity during school physical education classes was not significantly associated with aerobic capacity.

### 3.6. Multivariable Associations with Aerobic Capacity Score

A multivariable linear regression model was constructed to examine associations between PAL and the aerobic capacity category score. Aerobic capacity was operationalized as a three-level ordered classification (Needs Improvement, Acceptable, Outstanding), coded as an ordinal score (1–3). Age, sex, and BMI were included as covariates based on their established relationships with adolescent physical fitness. Results are presented in Table 5.

The model demonstrated a significant overall fit (F(4;107) = 8.45, *p* < 0.001) and explained 24% of the variance in aerobic capacity (adjusted R^2^ = 0.21). Physical activity level was positively associated with aerobic capacity (B = 0.22; 95% CI: 0.05–0.38; *p* = 0.010). Age also showed a positive association (B = 0.09; 95% CI: 0.01–0.18; *p* = 0.032), whereas sex was inversely associated with aerobic capacity (B = −0.39; 95% CI: −0.63 to −0.16; *p* < 0.001). BMI was not independently associated with aerobic capacity after adjustment (B = −0.04; 95% CI: −0.08 to 0.00; *p* = 0.080).

### 3.7. Exploratory Fitness Profiles Across Physical Activity and BMI

**Figure 2:**
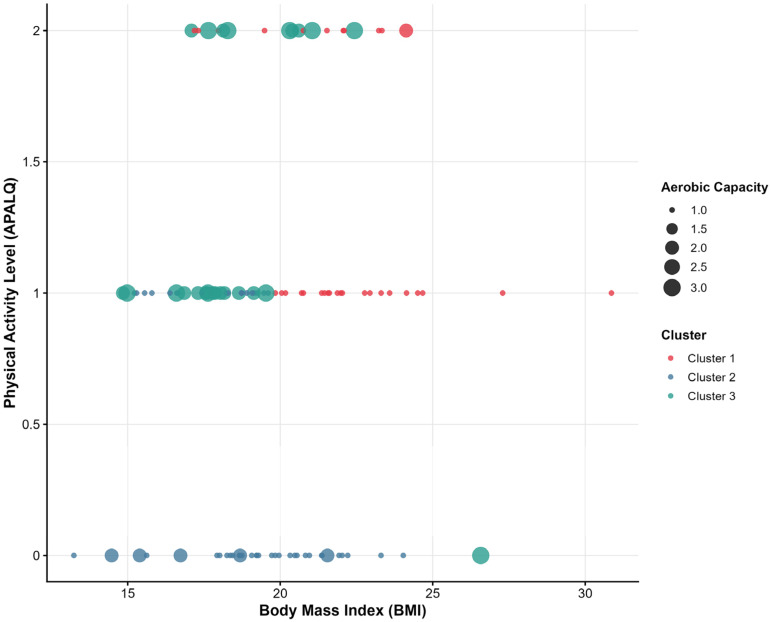
Exploratory fitness profiles according to PAL and BMI. Scatter plot derived from an exploratory unsupervised k-means clustering analysis using standardized variables (mean = 0; SD = 1). BMI is displayed on the horizontal axis, and APALQ on the vertical axis. Points are color-coded according to the identified clusters: red (Cluster 1), blue (Cluster 2), and turquoise/green (Cluster 3). Point size is proportional to aerobic capacity, with larger markers indicating higher values.

To examine the joint configuration of BMI, PAL and aerobic capacity, an exploratory unsupervised k-means clustering analysis was conducted following variable standardization. A three-cluster solution, selected based on the elbow criterion, is presented in Figure 2.

The observed distribution highlights distinct multivariate profiles within the adolescent population. One cluster primarily comprised sedentary individuals with lower levels of aerobic capacity across the BMI continuum. A second profile grouped adolescents with higher PAL and generally better cardiorespiratory fitness. A third cluster included participants with higher BMI values who exhibited variability in aerobic capacity despite reporting comparable activity levels.

Taken together, these patterns indicate a heterogeneous population structure in which aerobic capacity is not uniformly distributed among adolescents with similar levels of physical activity. This approach provides a structural perspective that complements the inferential findings and should be interpreted as exploratory and descriptive.

## 4. Discussion

This study examined the association between PAL, BMI and aerobic capacity category score in adolescents from a peri-urban setting. The findings indicate that PAL was independently associated with higher aerobic capacity categories after adjustment for age, sex, and BMI. This pattern is consistent with previous multivariable research, including the AVENA and ActiveBrains projects, which reported attenuation of anthropometric associations when behavioral variables were incorporated into statistical models [22,23].

From a physiological standpoint, this pattern aligns with established evidence indicating that habitual physical activity promotes central and peripheral adaptations—such as improved stroke volume, enhanced oxidative metabolism, and increased capillary density—that are reflected in better performance on progressive shuttle-run tests [24]. Although VO_2_max was not directly measured in the present study, the aerobic capacity categories derived from the 20 m shuttle run test represent a functional proxy of cardiorespiratory fitness. Within this interpretative framework, the observed association suggests that regular motor stimulation may be more closely aligned with aerobic performance than anthropometric classification alone.

The positive relationship between PAL and aerobic capacity reinforces accumulated evidence linking sustained movement behavior to cardiovascular adaptation during adolescence [25]. Rather than reflecting body mass per se, aerobic performance appears to mirror the functional responsiveness to habitual physical activity, supporting the interpretation of cardiorespiratory fitness as a behaviorally modifiable characteristic during this developmental stage.

In contrast, although descriptive analyses showed that most adolescents—regardless of BMI category—were classified within the “Needs Improvement” level, BMI did not independently explain variability in aerobic capacity after adjustment. This finding suggests that anthropometric classification may not adequately capture functional performance during adolescence. BMI is an indirect indicator that does not differentiate between fat mass and lean mass; therefore, its explanatory capacity regarding physiological performance is inherently limited. In Latin American contexts characterized by the coexistence of underweight and overweight, body mass alone may not reliably reflect functional status. Previous research indicates that adolescents with lower body mass may demonstrate favorable aerobic performance due to reduced mechanical load, whereas excess adiposity may impose additional energetic demands without necessarily determining aerobic responsiveness [26].

Sex differences were consistent across both PAL distribution and aerobic capacity categories. Girls showed a higher prevalence of sedentary behavior and were more frequently classified in lower aerobic capacity categories. In the adjusted model, sex remained independently associated with aerobic capacity. Although sociocultural and environmental determinants were not directly assessed, these patterns are congruent with literature describing gender disparities in adolescent physical activity participation. The ASSO project, for example, identified female sex as a determinant of lower physical fitness levels, associated with perceived barriers such as fatigue, low motivation, and body image concerns [22]. These findings highlight the need to interpret functional performance within broader social contexts.

Exploratory cluster analysis further revealed heterogeneous configurations combining PAL, BMI and aerobic capacity. Adolescents with comparable activity levels did not exhibit uniform functional performance profiles, suggesting that aerobic capacity emerges from the interplay of behavioral and anthropometric factors rather than from a single determinant. However, given the descriptive and exploratory nature of the clustering approach, these findings should be interpreted cautiously. Similar multivariate profiles—such as the so-called “Fat but Fit” or “Fat & Strong” configurations—have been described in prior research, emphasizing that functional capacity does not derive linearly from body mass classification [27]. Such configurations support the notion that functional status does not derive linearly from anthropometric category.

The peri-urban context in which this study was conducted provides an additional interpretative dimension. Transitional territories between rural and urban systems may modify access to recreational spaces, patterns of mobility, and opportunities for structured or informal physical activity [28]. Although environmental variables were not directly modeled, situating the findings within a peri-urban setting allows interpretation through an ecological lens, where school infrastructure, neighborhood design, and territorial transition may influence movement behaviors. Evidence suggests that availability of sports facilities and structured school-based programs can mitigate cardiovascular risk in contexts undergoing socio-environmental transformation [29].

From a public health perspective, these findings support prioritizing strategies that promote regular physical activity among adolescents rather than focusing exclusively on body weight classification. Given that PAL was independently associated with aerobic capacity, interventions centered solely on weight reduction may overlook clinically meaningful functional improvements achievable through movement promotion. Strengthening sustained opportunities for physical activity within schools and communities may represent a pragmatic approach to enhancing cardiorespiratory fitness in populations characterized by heterogeneous nutritional profiles.

Several strengths support the interpretability of the findings, including the use of a validated instrument for physical activity assessment, standardized field testing for aerobic capacity classification, and multivariable modeling that accounted for key demographic factors. Additionally, the exploratory clustering analysis provided a structural perspective on population heterogeneity, complementing inferential results.

Nevertheless, important limitations must be acknowledged. The cross-sectional design precludes causal inference, and the observed associations should not be interpreted as directional effects. The use of a non-probabilistic sample may limit generalizability to other peri-urban populations. Physical activity was assessed via self-report, introducing potential recall and social desirability bias despite acceptable reliability indices. Furthermore, potential clustering at the classroom or institutional level was not explicitly modeled and may have influenced variance estimates. Longitudinal designs incorporating objective physical activity measures and environmental indicators would allow more precise characterization of the dynamic relationships observed.

## 5. Conclusions

Aerobic capacity in adolescents was independently associated with PAL but not with the body mass index after adjustment for age and sex. These findings indicate that functional performance in this population is more closely aligned with behavioral patterns than with anthropometric classification alone.

Given that adolescence represents a critical stage for the consolidation of health-related behaviors, promoting sustained engagement in physical activity remains central to strengthening functional health indicators. The periurban context offers an additional interpretative layer, as environments undergoing territorial transition may shape both movement opportunities and lifestyle configurations.

Longitudinal research incorporating objective measures and environmental indicators is warranted to clarify the temporal structure of these associations and to inform context-sensitive strategies for adolescent health promotion.

## Figures and Tables

**Table 1:** Descriptive Analysis of Sociodemographic and Anthropometric Variables.

Characteristics	Total Population (*n* = 112)	Females (*n* = 53)	Males (*n* = 59)	*p* Value
Age (years), Mean ± SD	14.70 ± 1.29	14.73 ± 1.37	14.67 ± 1.23	0.6840
Age (years) Median (Q1–Q3)	15 (14–16)	15 (14–16)	15 (14–15.5)	
Age (years) Min–Max	(12–17)	(12–17)	(12–17)	
Grade Level, *n* (%)	112 (100)	53 (47.3)	59 (52.7)	
8th grade	36 (32.14)	12 (10.71)	24 (21.43)	
9th grade	36 (32.14)	14 (12.5)	22 (19.64)	
10th grade	14 (12.5)	8 (7.14)	6 (5.36)	
11th grade	26 (23.21)	19 (16.96)	7 (6.25)	
Weight (kg), Mean ± SD	51.77 ± 8.83	50.46 ± 7.48	52.95 ± 9.81	0.1380
Height (cm), Mean ± SD	162.10 ± 8.74	157.45 ± 6.20	166.28 ± 8.61	**<0.001**
BMI (kg/m^2^), Mean ± SD	19.67 ± 2.82	20.33 ± 2.58	19.08 ± 2.92	**0.0041**
BMI *				
Thinness *n* (%)	13 (11.61)	4 (7.55)	9 (15.25)	
Risk of thinness *n* (%)	12 (10.71)	3 (5.66)	9 (15.25)	
Normal Weight *n* (%)	79 (70.54)	41 (77.36)	38 (64.41)	
Overweight *n* (%)	7 (6.25)	5 (9.43)	2 (3.40)	
Obesity *n* (%)	1 (0.89)	0 (0)	1 (1.69)	

BMI *: The classification will be determined based on weight and height-for-age tables established by Resolution 2465 of 2016, issued by the Colombian Government. Group differences were assessed as follows: Age and BMI with Wilcoxon–Mann–Whitney; Weight and Height with Student’s *t*-test assuming equal variances; Grade level with Chi-square (2 × 4); BMI classification with Chi-square using permutation-based *p*-values (two-tailed, α = 0.05-(*p* < 0.05)). Bold values indicate statistically significant differences (*p* < 0.05).

**Table 2:** Distribution of students across APALQ categories by sex.

APALQ/Sex	Total *n* = 112	Female *n* = 53 (95% CI)	Males *n* = 59 (95% CI)	*p* Value
Sedentary	36 (32.14)	26 (49.1) (36.1–62.1)	10 (16.9) (9.5–28.5)	**<0.001**
Moderately active	56 (50.00)	19 (35.8) (24.3–49.3)	37 (62.7) (50.0–73.9)	**0.004**
Very active	20 (17.86)	8 (15.1) (7.9–27.1)	12 (20.3) (12.0–32.3)	0.469

The overall distribution differed between sexes (χ^2^(2) = 13.41, *p* = 0.001). Pairwise proportion tests (two-sided) showed higher sedentary prevalence in females (*p* < 0.001) and higher moderate activity in males (*p* = 0.004); no sex difference was observed for very active (*p* = 0.469). Values are % with 95% CIs. Bold indicates significant differences (*p* < 0.05).

**Table 3:** Comparison of Aerobic Capacity Categories by Sex (Course-Navette).

Aerobic Capacity/Sex	Males *n* (%)	Females *n* (%)	*n* (%)	*p* Value
Needs improvement	32 (54.24)	49 (92.5)	81 (72.3)	**<0.001**
Acceptable	18 (30.5)	3 (5.7)	21 (18.8)	**<0.001**
Outstanding	9 (15.3)	1 (1.9)	10 (8.9)	**0.0133**

Global χ^2^(2) = 20.42, *p* < 0.001. Pairwise sex differences: two-sided z-tests for proportions with Wilson 95% CIs. Bold indicate significant differences (*p* < 0.05).

**Table 4:** Distribution of aerobic capacity in relation to BMI among adolescents.

Aerobic Capacity	Underweight *n* (%)	Risk of Underweight *n* (%)	Normal Weight *n* (%)	Overweight *n* (%)	Obesity *n* (%)	Total *n* (%)
Needs improvement	6 (7.4)	6 (7.4)	62 (76.5)	6 (7.4)	1 (1.2)	81 (72.3)
Acceptable	3 (14.3)	3 (14.3)	12 (57.1)	3 (14.3)	0 (0)	21 (18.8)
Outstanding	1 (10.0)	3 (30.0)	5 (50.0)	1 (10.0)	0 (0)	10 (8.9)
Total	10 (8.9)	12 (10.7)	79 (70.5)	10 (8.9)	1 (0.9)	112 (100)

**Table 5:** Multivariable linear regression model examining factors associated with the aerobic capacity ordinal score.

Predictor	B *	95% CI	*p* Value
Intercept	1.08	−0.31 to 2.48	0.127
BMI	−0.04	−0.08 to 0.00	0.080
Physical activity	0.22	0.05 to 0.38	**0.010**
Age	0.09	0.01 to 0.18	**0.032**
Sex	−0.39	−0.63 to −0.16	**<0.001**

B * = unstandardized regression coefficient. The dependent variable was the aerobic capacity ordinal score (coded 1–3). The ordinal score was treated as an approximately continuous outcome. Model adjusted for age, sex, BMI, and physical activity level. Bold values indicate statistically significant differences (*p* < 0.05).

## Data Availability

The data presented in this study may be available on request from the corresponding author due to privacy and ethical restrictions.
